# Sodium Hydrosulphide Alleviates Remote Lung Injury Following Limb Traumatic Injury in Rats

**DOI:** 10.1371/journal.pone.0059100

**Published:** 2013-03-19

**Authors:** Jiaolin Ning, Liwen Mo, Hongzhi Zhao, Kaizhi Lu, Xinan Lai, Xianghong Luo, Hailin Zhao, Daqing Ma

**Affiliations:** 1 Department of Anesthesiology, Southwest Hospital, Third Military Medical University, Chongqing, China; 2 Department of Nephrology, People’s Liberation Army Chengdu Military Area Command General Hospital, Chengdu, Sichuan, China; 3 Department of hepatobiliary surgery, Xinqiao Hospital, Third Military Medical University, Chongqing, China; 4 State Key Laboratory of Trauma and Burns, Surgery Research Institute, Department of Traumatic Surgery, Daping Hospital, Third Military Medical University, Chongqing, China; 5 Anaesthetics, Pain Medicine and Intensive Care, Department of Surgery and Cancer, Faculty of Medicine, Imperial College London, Chelsea and Westminster Hospital, London, United Kingdom; Massachusetts General Hospital, United States of America

## Abstract

Hydrogen sulphide (H_2_S) was found to attenuate ventilator or oleic acid induced lung injury. The aim of this study was to explore the effects of exogenous H_2_S donor, sodium Hydrosulphide (NaHS), on lung injury following blast limb trauma and the underlying mechanisms. For *in vitro* experiments, pulmonary micro-vessel endothelial cells (PMVECs) were cultured and treated with NaHS or vehicle in the presence of TNF-α. For *in vivo*, blast limb traumatic rats, induced by using chartaceous electricity detonators, were randomly treated with NaHS, cystathionine gamma-lyase inhibitor (PAG) or vehicle. *In vitro*, NaHS (100 µM) treatment increased PMVECs viability and decreased LDH release into culture media after tumor necrosis factor (TNF) α challenge. In addition, NaHS treatment prevented the increase of nitric oxide, Intercellular Adhesion Molecule 1(ICAM-1) and interleukin (IL)-6 production and inducible nitric oxide synthase activation induced by TNF-α. Knock-down of NF-E2-Related Factor 2 (Nrf2) partially abolished the protective effect of NaHS. *In vivo*, NaHS treatment significantly alleviated lung injury following blast limb trauma, demonstrated by a decreased histopathological score and lung water content. Furthermore, NaHS treatment reversed the decrease of H_2_S concentration in plasma, prevented the increase of TNF-α, IL-6, malondialdehyde and myeloperoxidase, increased the Nrf2 downstream effector glutathione in both plasma and lungs, and reversed the decrease of superoxide dismutase in both plasma and lungs induced by blast limb trauma. Our data indicated that NaHS protects against lung injury following blast limb trauma which is likely associated with suppression of the inflammatory and oxidative response and activation of Nrf2 cellular signal.

## Introduction

Limb traumatic injury induced by blast is one of the common injurious forms in military conflict and terrorist attacks [1], [2], [3], [4]. Blast limb trauma is not only manifested as regional damage, but also induces remote organ injury, such as remote lung injury as reported in our previous study [5]. Remote lung injury poses a big challenge in clinical management of blast limb trauma. Prompt medical interference is, therefore, necessary to avoid the exacerbation of acute lung injury and achieve good prognosis of the wounded patients.

It was found that remote lung injury following blast limb trauma was accompanied with not only inflammatory response and oxidative stress, but also depression of cystathionine γ-lyase (CSE)/hydrogen sulphide (H_2_S) system [5]. Like carbon monoxide (CO) and nitric oxide (NO), H_2_S has been considered as the third member of gaseous molecules with potent bio-physiological properties [6]. Endogenous H_2_S is produced from cysteine or cysteine with homocysteine by cystathionine γ-lyase (CSE) in the peripheral tissues such as the lungs and cystathionine β-synthase (CBS) in the central nervous system [6]. Endogenous H_2_S plays an important role of physiological regulatory function in cardiovascular and nervous system [7]. In addition, it was reported that H_2_S exerts anti-inflammatory, anti-oxidative and anti-apoptotic effects [8], [9]. Recently exogenous H_2_S gas inhalation or H_2_S donor administration was reported to alleviate ventilator [10], [11] or oleic acid [12] induced acute lung injury. In our previous study, at the early stage of blast limb trauma, plasma H_2_S was found to be reduced and lung CSE activity was shown to depress as well [5]. All those studies indicated that exogenous H_2_S donor or H_2_S may have a therapeutic value in acute lung injury. The aim of the present study was to explore the potential protective effects of Sodium Hydrosulphide (NaHS), exogenous H_2_S donor [8] on remote lung injury and the underlying mechanism in a rat model of blast limb trauma.

## Results

### NaHS Protected Pulmonary Micro-vascular Endothelial Cells (PMVECs) against Injury Induced by TNF-α via Transcription Factor NF-E2-Related Factor 2 (Nrf2)

**Figure 1:**
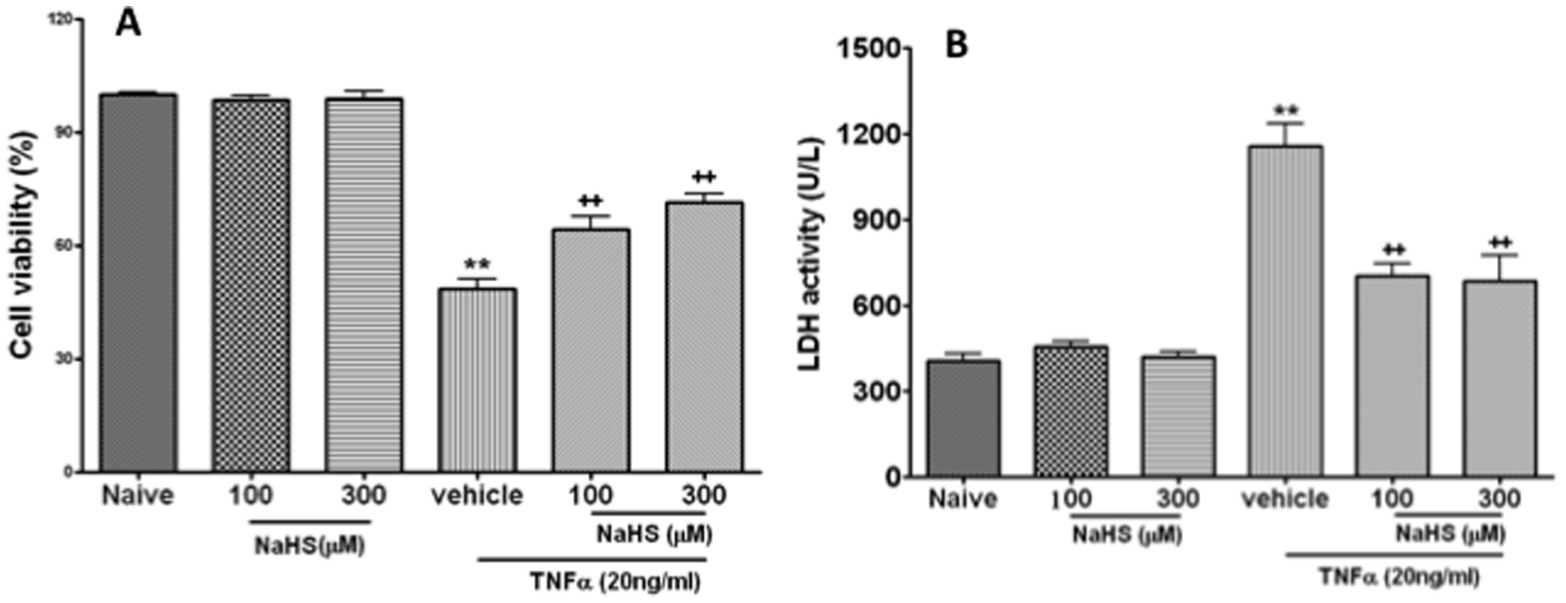
Sodium Hydrosulphide (NaHS) attenuates the pulmonary micro-vessel endothelial cells injury induced by tumour necrosis factor alpha (TNF-α). The cells viability is measured with 3-(4, 5-Dimethyl-2-thiazolyl)-2, 5- diphenyl-2H-tetrazolium bromide assay (MTT) assay with TNF-α with or without NaHS (A). The injured cells indicated with lactate dehydrogenase release in the culture media with TNF-α treatment in the presence or absence of NaHS (B). Data are mean ± SEM (n = 12); ** p<0.01 *vs* Naïve,++p<0.01 *vs* vehicle.

Viability of PMVECs treated with TNF-α at 20 ng/ml was decreased, which was demonstrated by MTT assay and increase of LDH activity, the viability of PMVECs treated with 100 µM (64±10%) or 300 µM (71±7%) was increased compared to PMVECs treated with vehicle (49±7%) after TNF-α (20 ng/ml) exposure (p<0.01) (Fig. 1A). NaHS treatment at 100 µM or 300 µM decreased LDH activity from 1157±229 U/ml to 704±118 U/ml and 688±248 U/ml after TNF-α (20 ng/ml) exposure, respectively (p<0.01) (Fig. 1B). Therefore there was no significant difference between 100 µM NaHS treatment or 300 µM NaHS treatment plus TNF-α (Fig. 1A, B). NaHS at 100 µM or 300 µM alone had no obvious effects on the PMVECs viability and LDH activity (Fig. 1A, B). The NaHS at 100 µM was selected in the following experiments.

**Figure 2:**
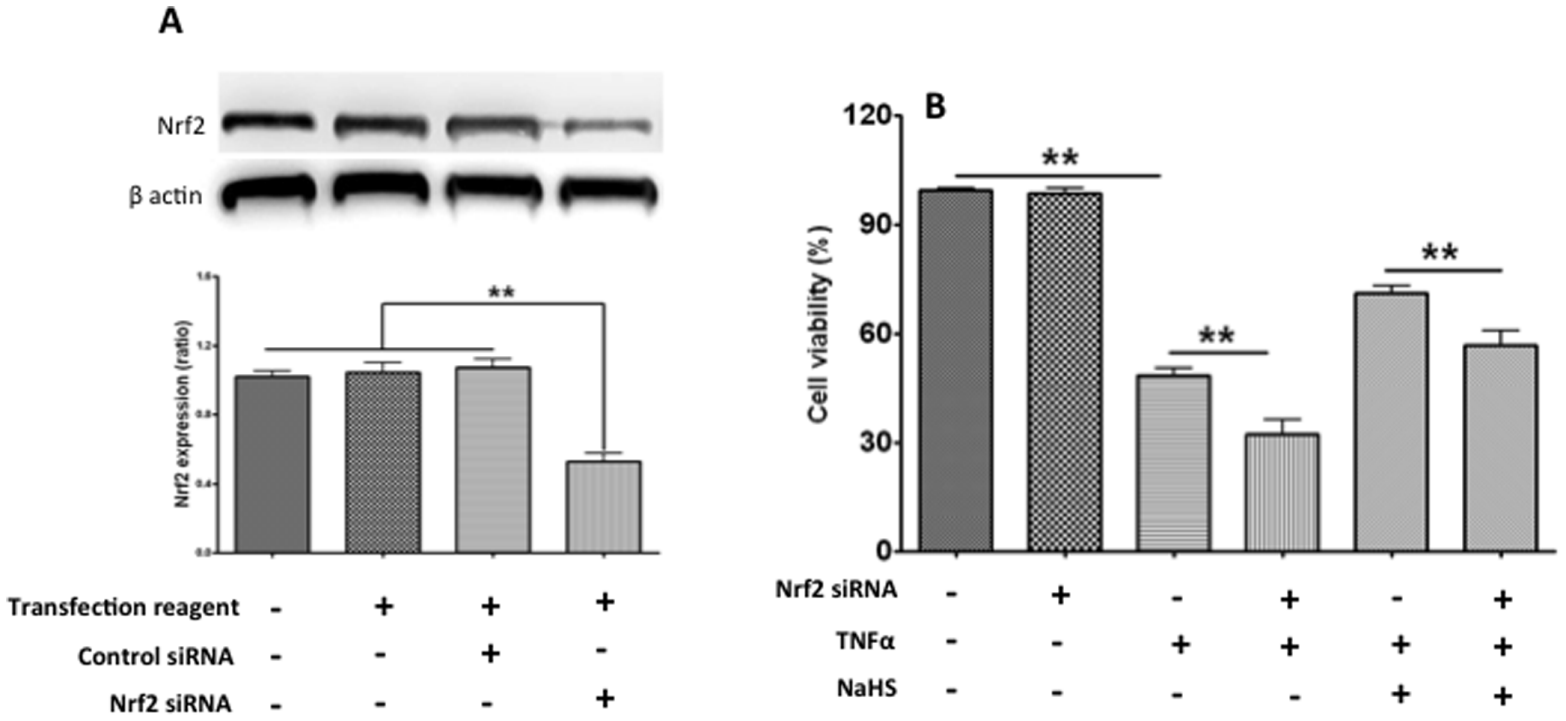
The protective effect of Sodium Hydrosulphide (NaHS) via Nrf2 *in vitro*. Nrf2 knockdown with siRNA [upper panel: example bands of Nrf2 and internal control of housekeeping protein β-actin; bottom panel: mean data (mean ± SEM; n = 3) of Nrf2 normalised with β-actin] in the cultures of pulmonary micro-vessel endothelial cells (A). The viability of pulmonary micro-vessel endothelial cells with or without knockdown of Nrf2 was determined with 3-(4, 5-Dimethyl-2-thiazolyl)-2, 5- diphenyl-2H-tetrazolium bromide (MTT) assay in the presence or absence of TNF-α/NaHS (B). Data are mean ± SEM (n = 12); ** p<0.01.

Nrf2 is a key transcription factor that regulates antioxidant response element (ARE) -mediated expression of antioxidant enzyme and cyto-protective proteins. In order to analyse the role of Nrf2 on the NaHS protection, Nrf2 siRNA was employed to down-regulate Nrf2 protein expression by about 50% (Fig. 2A). Although Nrf2 siRNA alone did not reduce cell viability, TNF-α caused a decrease of cell viability which was further reduced by siRNA Nrf2 (48±6% vs 32±12%, p<0.001). Nrf2 siRNA partially abolished the protection of NaHS (71±6% vs 57±12%, p<0.01) (Fig. 2B).

### NaHS Treatment Decreased Nitric Oxide (NO) Production Induced by TNF-α via Suppression of Inducible Nitric Oxide Synthase (iNOS)

**Figure 3:**
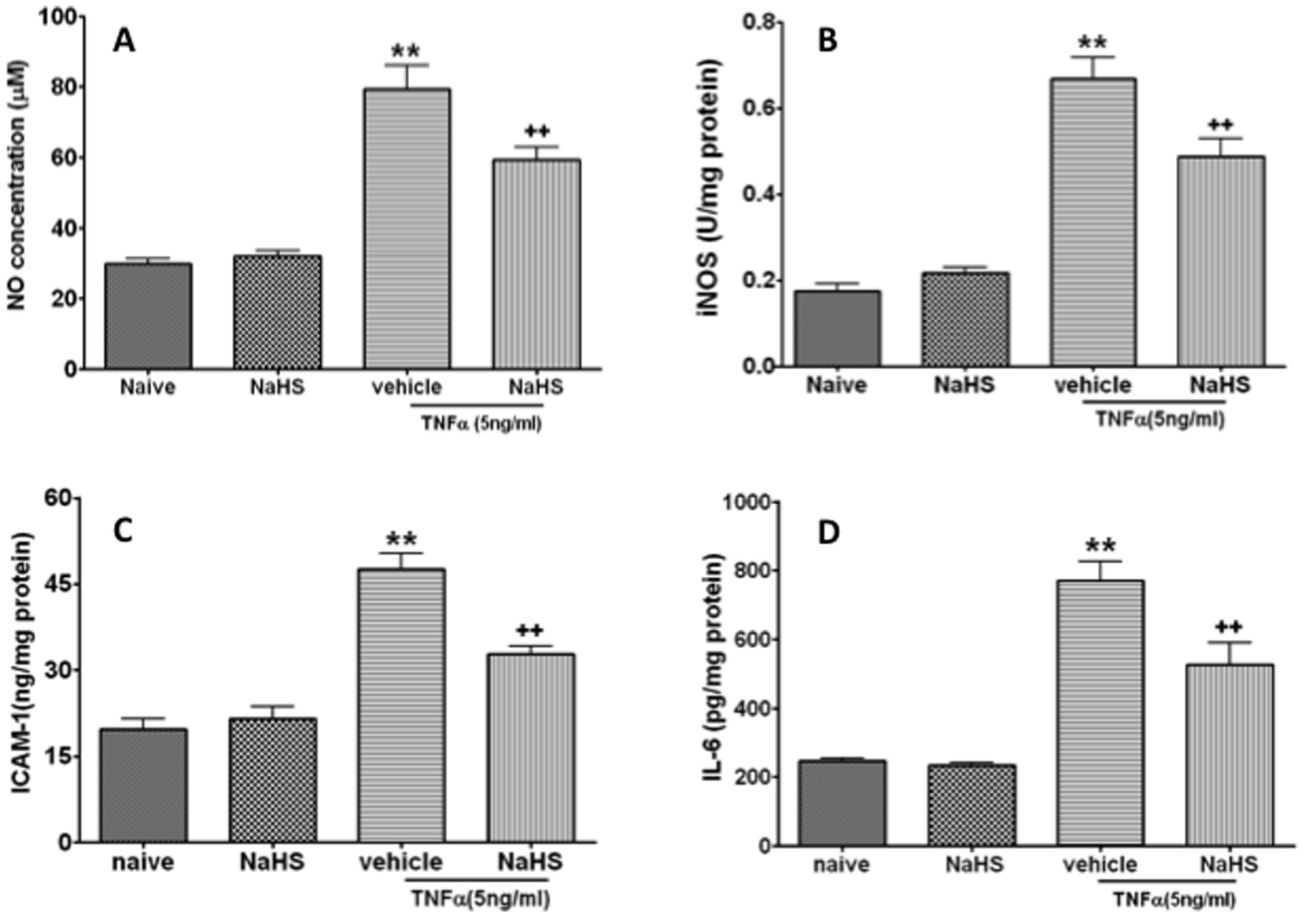
NaHS suppresses the productions of oxidative and inflammatory mediators in pulmonary micro-vessel endothelial cells induced by TNF-α. Nitric oxide concentration (A), Inductive nitric oxide synthase (B), Intercellular adhesion molecule (ICAM) 1 (C) and interleukin (IL) 6 (D). Data are mean ± SEM (n = 12); ** p<0.01 *vs* Naïve,++p<0.01 *vs* vehicle.

Excessive production of NO induced by NO synthase (NOS) plays a critical role in the pathogenesis of acute lung injury and PMVECs are considered as one of source of excessive NO [13], therefore NO concentration and iNOS activity were determined in this study. TNF-α treatment at 5 ng/ml, which has no effect on cell viability, induced a significant increase of NO concentration compared to naïve control (29.8±4.1 µM vs 79.4±18.6, p<0.01) (Fig. 3A), NaHS treatment prevented this increase of NO concentration induced by TNF-α (79.4±18.6 µM to 59.3±10.3, p<0.01) (Fig. 3A). TNF-α treatment induced a significant increase of iNOS activity compared to naïve control (0.17±0.05 U/mg protein *vs* 0.67±0.14, p<0.01) (Fig. 3B), and NaHS treatment partially reversed the increase of iNOS activity induced by TNF-α (0.67±0.14 U/mg protein *vs* 0.49±0.12, p<0.01) (Fig. 3B). NaHS treatment alone at 100 µM has no significant effect on NO concentration and iNOS activity.

### NaHS Treatment Decreased the Expression of Intercellular Adhesion Molecular 1 (ICAM1) and Interleukin (IL) 6 Induced by TNF-α

TNF-α up-regulated the expression of ICAM1 compared to naïve control (47.5±8.1 ng/mg protein *vs* 19.7±5.2, p<0.01) (Fig. 3C), and NaHS treatment partially reversed this increase induced by TNF-α (32.8±4.0 ng/mg protein *vs* 47.5±8.1, p<0.01) (Fig. 3C). TNF-α treatment up-regulated the expression of IL-6 in the PMVECs compared to naïve control (769±161 pg/mg protein *vs* 247±19, p<0.01) (Fig. 3D); NaHS treatment prevented this increase induced by TNF-α (526±180 pg/mg protein *vs* 769±161, p<0.01) (Fig. 3D). Treatment with NaHS alone did not significantly change ICAM1 and IL-6 expression (p>0.05) (Fig. 3C, D).

### NaHS Treatment Alleviated Lung Injury Induced by Blast Limb Trauma

**Figure 4:**
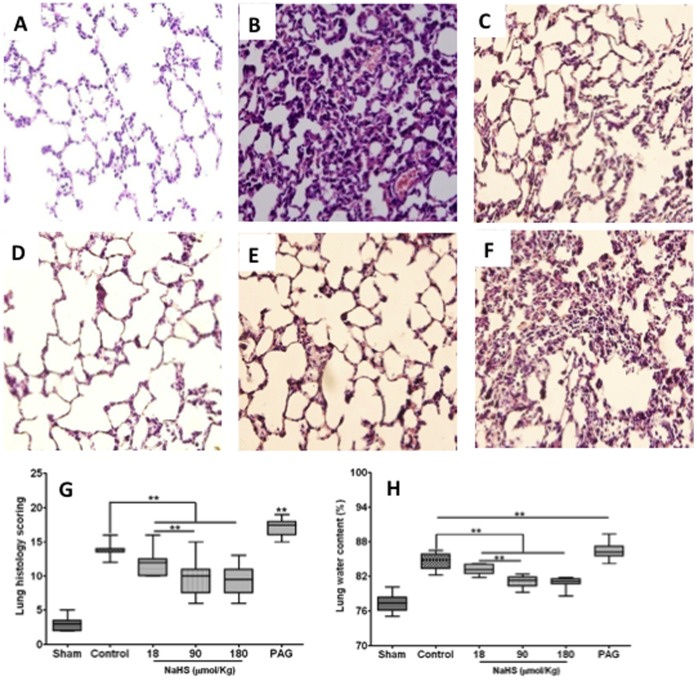
The effects of NaHS or DL- propargylglycine (PAG) treatment on the lung histopathology changes and oedema induced by blast limb trauma. Representative microphotographs were taken from Sham (A), Control (B), 18 µmol/Kg (C), 90 µmol/Kg (D), 180 µmol/Kg NaHS treatment (E), PAG (50 mg/Kg) (F). Pathohistological scoring data of lung injury were presented in a box-whisker plot (the boxes are constructed with 25% and 75% confident intervals, median and maximum or minimum individual values) (G). Lung water content (H). Data are mean ± SEM (n = 8); ** p<0.01.

In contrast to the micro-structure of the lungs in the naïve control (Fig. 4A), blast limb trauma induced alveolar congestion, hemorrhage, breakdown of alveoli architecture, alveolar wall thickening and cell infiltration (Fig. 4B). NaHS treatment at 18 µmol/Kg, 90 µmol/Kg and 180 µmol/Kg alleviated the morphology changes mentioned above (Fig. 4C, D, E). Whereas PAG treatment exacerbated lung histopathological changes induced by blast limb trauma (Fig. 4F). These changes are demonstrated in the box-and-whisker plot of the scoring data (Fig. 4G), showing that NaHS treatment at 180 µmol/Kg failed to further alleviate the morphology changes compared with NaHS at 90 µmol/Kg (Fig. 4D, E, G). This “ceiling effect” was also confirmed with the lung water content (Fig. 4H). Owing to this ceiling effect, a single dose of NaHS at 90 µmol/Kg was used for the most further studies with the results described below.

### NaHS Treatment Reversed the Decrease of Plasma H_2_S Induced by Blast Limb Trauma

**Figure 5:**
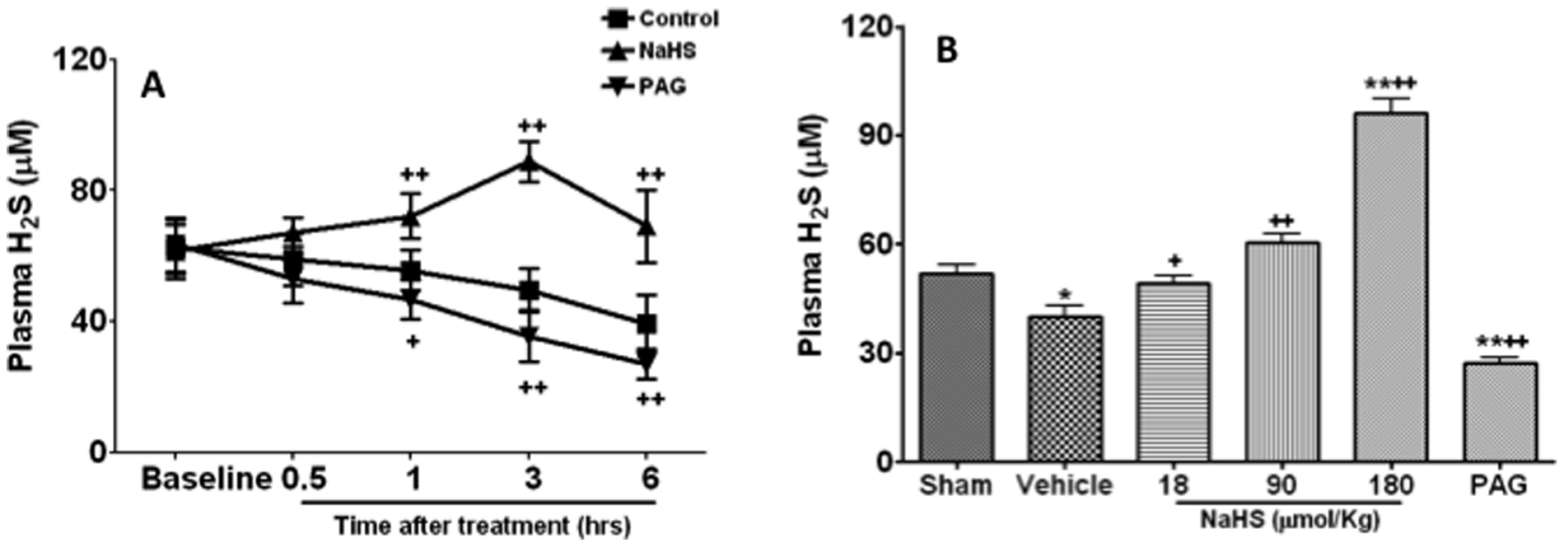
The changes of plasma hydrogen sulphide (H_2_S) in blast limb trauma rats after treatment with NaHS or DL- propargylglycine (PAG). The time course of plasma H_2_S after treatment with vehicle, NaHS (90 µmol/Kg) and PAG (50 mg/Kg) (A); Plasma H_2_S at 6 hrs after treated with vehicle, NaHS (18, 90 or 180 µmol/Kg) and PAG (50 mg/Kg) (B). *P<0.0.5, ** p<0.01 *vs* Sham;+p<0.05,++p<0.01 *vs* control.

Blast limb trauma resulted in decrease of plasma H_2_S in a time-dependent manner after blast (Fig. 5A). NaHS (90 µmol/kg) treatment reversed this decrease, and plasma H_2_S level reached to the peak at 3 hrs (88.7±6.2 µM) after NaHS treatment and returned to baseline at 6 hrs (60.4±6.8 µM) after NaHS treatment (Fig. 5A). PAG treatment further exacerbated the decrease of plasma H_2_S induced by blast limb trauma (Fig. 5A). To explore the effects of different doses of NaSH treatment on the plasma H_2_S, Plasma H_2_S was determined at 6 hrs after treatment (Fig. 5B). Plasma H_2_S was dose-dependently increased in the rats treated with NaHS at dose of 18 µmol/Kg (49.1±6.5, p<0.05), 90 µmol/Kg (60.4±6.8, p<0.01) and 180 µmol/Kg (96.0±11.2, p<0.01) compared to that (38.8±8.8 µM) in vehicle treated rats (Fig. 5B). PAG treatment further decreased plasma H_2_S concentration (27.1±4.6 µM) compared to vehicle (38.8±8.8 µM, p<0.01) (Fig. 5B).

### Physiological Parameters

**Figure 6:**
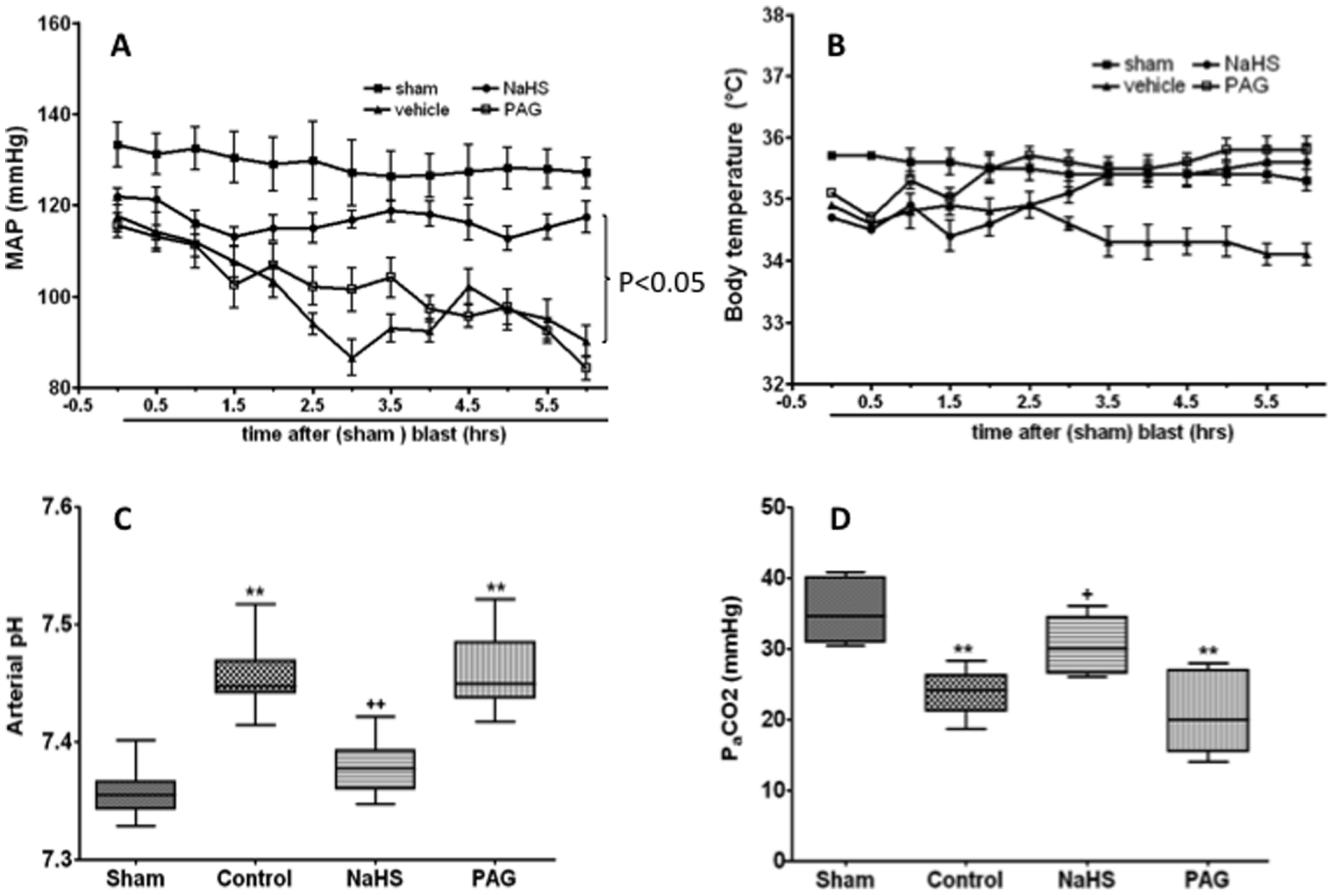
Physiological parameters during the course of study. Mean data of mean artery pressure (A), body temperature (B), arterial pH (C) and P_a_CO_2_ (D). Sham: the same surgical procedure but without blast and any treatments; Vehicle: blast+saline administration; NaHS: blast +90 µmol/Kg NaHS in saline; DL- propargylglycine (PAG): blast +50 mg/Kg PAG in saline. Data are presented as mean ± SEM (n = 8).

NaHS treatment partially reversed the decrease of artery blood pressure induced by blast limb trauma, PAG treatment had no significant effect on the artery blood pressure in rats underwent blast limb trauma compared to vehicle treatment (Fig. 6A). NaHS or PAG treatment led to a slight increase of body temperature, but there was no significant difference when compared with that of rats in the vehicle group (Fig. 6B). NaHS treatment (90 µmol/Kg) corrected abnormal pH and P_a_CO_2_ induced by trauma to within the normal physiological range but PAG abolished its effect (Fig. 6C and D). P_a_O_2_ value was maintained normal range (>80 mmHg ) in all groups.

The blood loss induced by blast limb trauma was 2.12±0.13 ml, 2.25±0.9 ml and 2.16±0.11 ml in the control, NaHS treatment (90 µmol/Kg) and PAG treatment group respectively. There was no statistical difference between groups.

### NaHS Treatment Suppressed Systemic and Local Inflammatory Reaction Induced by Blast Limb Trauma

**Figure 7:**
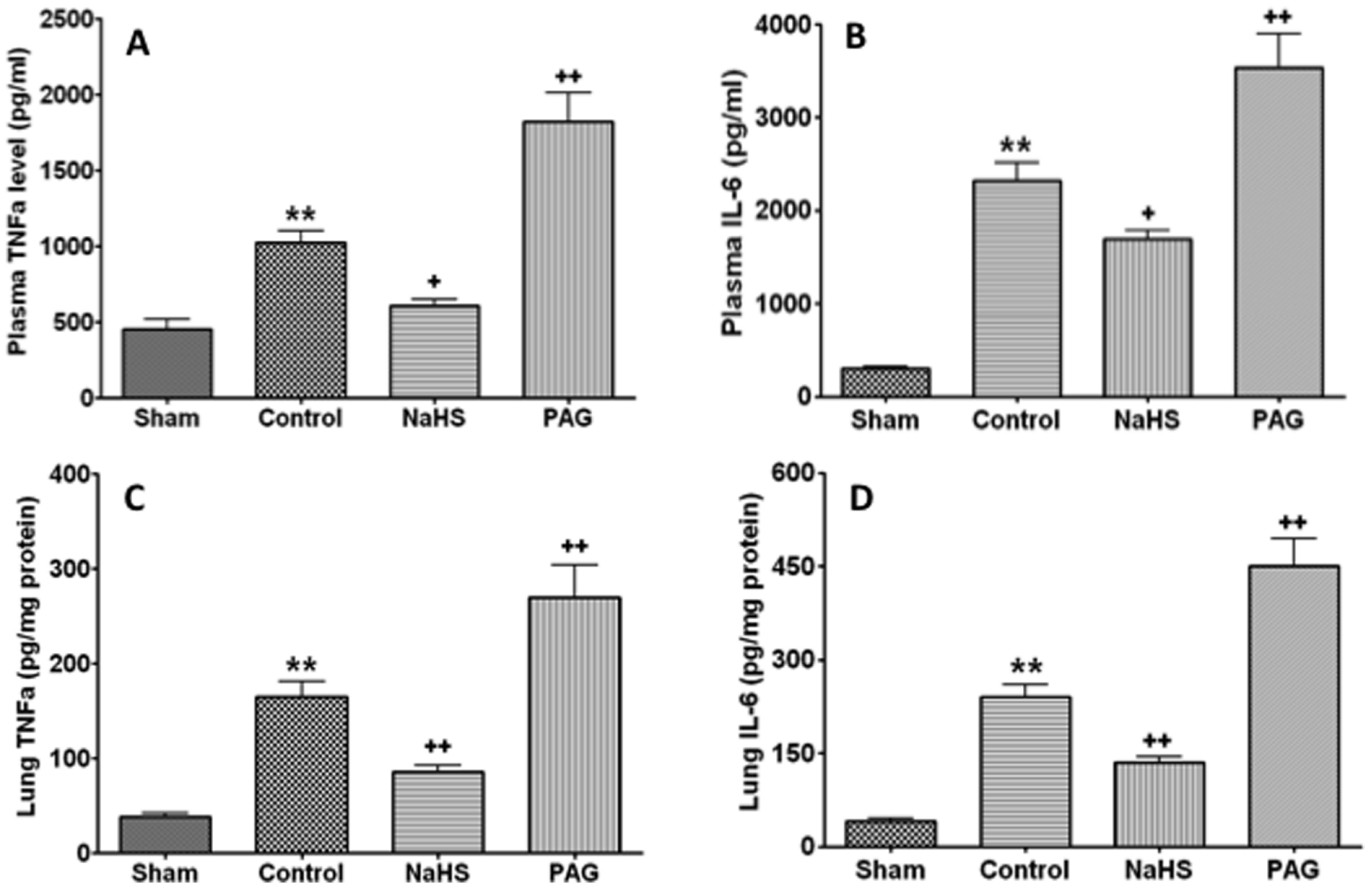
The effects of NaHS or DL- propargylglycine (PAG) treatment on cytokines in plasma and lungs after blast limb trauma . Plasma TNF-α (A), interleukin (IL)-6 (B), Lung TNF-α (C), IL- 6 (D). Data are mean ± SEM (n = 8).*P<0.0.5, ** p<0.01 *vs* Sham;+p<0.05,++p<0.01 *vs* control.

The plasma TNF-α and IL-6 concentration in the vehicle treated rats was 1083±160 pg/ml and 2481±311 pg/ml, respectively. Plasma TNF-α (611±116 pg/ml) and IL-6 (1697±238 pg/ml) concentration was decreased in the NaHS treated rats compared to the vehicle treated rats, respectively (p<0.01); Plasma TNF-α (1822±537 pg/ml) and IL-6 (3529±1057 pg/ml) was increased in PAG treated rats compared to vehicle treated rats, respectively (p<0.01) (Fig. 7A, B). A similar pattern of changes of TNF-α and IL-6 was noticed in the lungs (Fig. 7C, D).

### NaHS Treatment Suppressed Activation of Neutrophil Induced by Blast Limb Trauma

**Figure 8:**
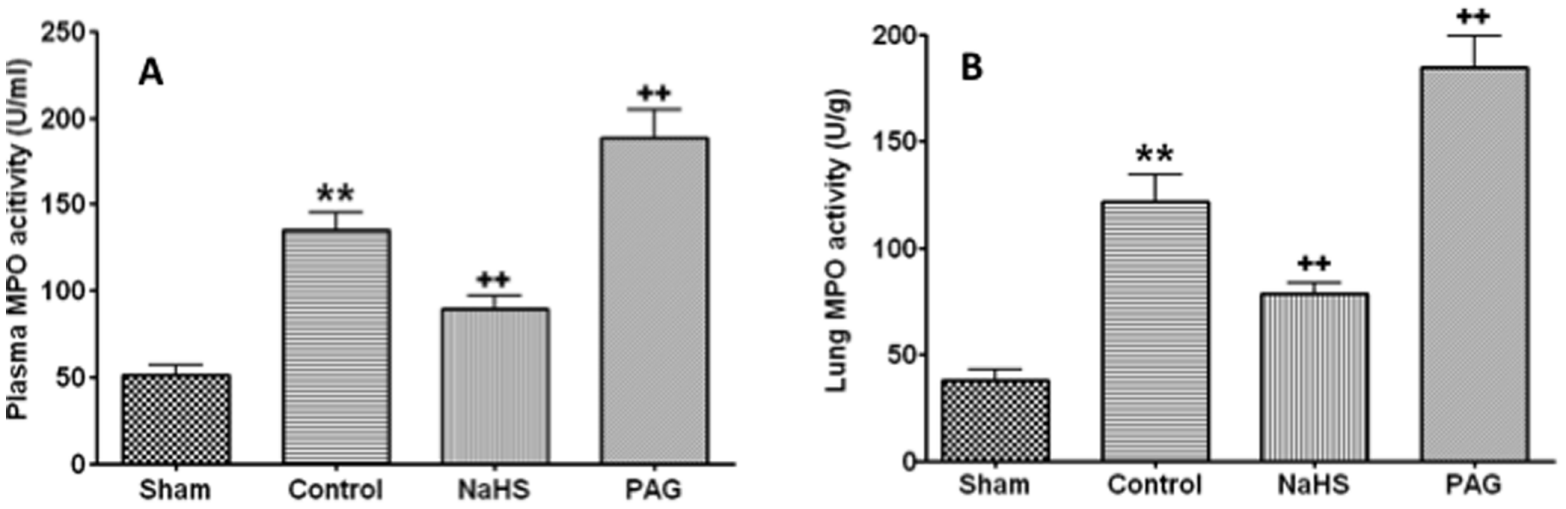
The effects of NaHS or DL- propargylglycine (PAG) treatment on myeloperoxidase (MPO) in plasma and lungs after blast limb trauma. Plasma MPO (A), Lung MPO (B). Data are mean ± SEM (n = 8);*P<0.0.5, ** p<0.01 *vs* Sham;+p<0.05,++p<0.01 *vs* control.

The activation of neutrophil was evaluated with myeloperoxidase (MPO) assay. Blast limb trauma induced an increase of MPO activity in both plasma (141.6±24.6 U/ml) and lung (127.5±34.1 U/g), and this increase was attenuated by NaHS treatment (plasma 89.3±21.4 U/ml; lung: 78.5±14.6 U/g, all p<0.01) (Fig. 8A, B). PAG treatment further enhanced MPO activity in plasma and lung in blast limb trauma rats (plasma: 188.3±47.5 U/ml; lung 184.9±41.1 U/g, all p<0.01) (Fig. 8A, B).

### NaHS Treatment Attenuated Oxidative Stress Induced by Blast Limb Trauma

**Figure 9:**
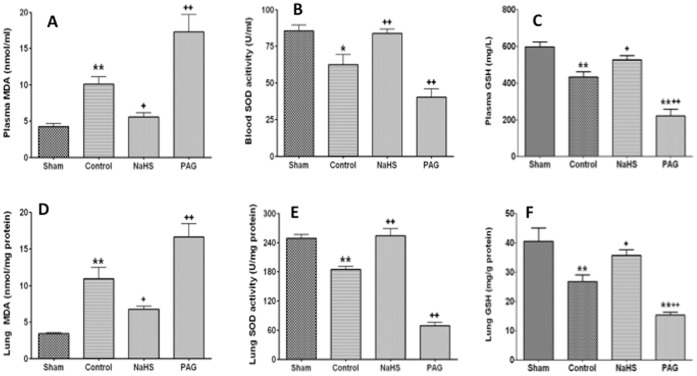
The effects of NaHS or PAG treatment on oxidative stress induced by blast limb trauma. Plasma mylondialdehyde (MDA) (A), Superoxide dismutase (SOD) in Plasma (B), GSH in plasma (C) Lung MDA (D), SOD in lungs (E), GSH in Lung (F). Data are mean ± SEM (n = 8). *P<0.0.5, ** p<0.01 *vs* Sham;+p<0.05,++p<0.01 *vs* control.

Malondialdehyde (MDA), a lipid peroxidation product, and an anti-oxidative enzyme superoxide dismutase (SOD) and Glutathione (GSH) both in plasma and lung tissue were measured to evaluate the severity of oxidative stress in this study. Blast limb trauma resulted in the increase of MDA concentration in plasma (11.1±2.4 nmol/ml) and decrease of SOD activity (58.5±16.9 U/ml) in plasma compared to sham control (plasma MDA: 3.9±1.3 nmol/ml; plasma SOD: 89.8±8.7 U/ml). Those changes were reversed by NaHS treatment (MDA: 5.6±1.6 nmol/ml, p<0.01; SOD: 83.9±8.0 U/ml, p<0.05). However PAG treatment exacerbated the oxidative stress reflected by plasma MDA and SOD in the blast limb trauma rats (plasma MDA 17.3±6.8 nmol/ml; plasma SOD: 40.4±15.3 U/ml; all p<0.01) (Fig. 9A, B). Blast limb trauma induced a decrease of plasma GSH (434.3±77.9 mg/L) compared with that of Sham rats (597.6±75.7 mg/L, p<0.01) (Fig. 9C). Plasma GSH was increased in the NaHS treated rats (526.3±68.0 mg/L) compared to the vehicle treated rats (434.3±77.9 mg/L, p<0.05). PAG treatment further exacerbated the decrease of plasma GSH induced by blast limb trauma (222.3±103.3 mg/L *vs* 434.3±77.9, p<0.05) (Fig. 9C). MDA concentration, SOD activity and GSH in the lungs show similar results (Fig. 9 D, E, F).

## Discussion

Traumatic injury like blast limb can trigger distant vital organ injury including the lungs. This “remote” lung injury following blast limb trauma is accompanied with systemic inflammatory and oxidative response and depression of cystathionine gamma-lyase (CSE)/H_2_S reported recently [5], which was also found in the LPS-treated mice [14]. The current study demonstrated that exogenous H_2_S donor, NaHS, attenuated lung injury following blast limb trauma, reversed the decrease of plasma H_2_S, suppressed cytokines release and accumulation of neutrophil, and inhibited oxidative stress induced by blast limb trauma. Our data further showed that lung injury, inflammatory response and oxidative stress in blast limb trauma were exacerbated by the administration of PAG, the CSE inhibitor of H_2_S production. Taken together, it can be concluded that the protection of NaHS against lung injury was through H_2_S gaseous signalling pathway. This conclusion is further supported by the following: 1) our *in vitro* data directly showed that NaHS *via* H_2_S in media prevented PMVECs injury induced by TNF-α; 2) Plasma H_2_S was decreased, which was in accordance with the changes of lung histology and water content; 3) Both *in vitro* and *in vivo* data indicated that cellular signal of Nrf2/GSH was activated by NaHS treatment. Furthermore, our conclusion was supported by a previous study in which inhaled H_2_S gas alleviated the ventilator induced lung injury [11].

It has been noted that the protective effects of NaHS are not correlated with the doses (18–180 µmol/kg) used in our study. This ceiling effect reported here has not been documented yet. Moreover, we found in our pilot studies that the toxic or lethal dose of NaHS was more than 360 µmol/kg or more than 1440 µmol/kg, respectively. This has been considered to be due to the inhibition of mitochondrial cytochrome enzymes and subsequently prevention of cellular respiration [15]. It should be pointed out that over-production of H_2_S may be harmful and also H_2_S plays an important role in the pathological process in some disease conditions. For example, plasma H_2_S was found to be increased in caerulein induced pancreatitis mice and H_2_S production was decreased by PAG of a CSE inhibitor showing to protect pancreatitis associated lung injury [16], [17]. Hence, the dual effects of H_2_S in the different pathological conditions should be recognised whilst maintaining homeostasis of H_2_S is important for normal organ function.

It is worth mentioning that plasma H_2_S was shown to decrease in a time-dependent manner after blast limb trauma. NaHS treatment at 90 µmol/Kg was found to increase the plasma H_2_S levels, and this increase reached to a peak level at 3 hours after treatment, which is not in line with the results reported previously [18]. The reasons of this discrepancy remain to be investigated but the different subject conditions (normal *vs* traumatic) is likely to be responsible to some extent. For example, CSE activity was found to decrease in traumatic rats [5] and this change may affect the time-course of H_2_S.

Blast limb trauma triggered the local and system inflammatory response, characterized by release of cytokines, such as TNF-α and IL-6, and those cytokines play important roles in the early phase of acute lung injury [5]. Indeed acute lung injury is considered as the result of systemic inflammatory reaction [19], [20]. In the current study, NaHS treatment was shown to significantly suppress the inflammatory reaction triggered by blast limb trauma, accompanied with the decrease of the lung water content and histological improvement. Blockage CES activity with a CSE inhibitor, PAG, further decreased the production of H_2_S in blast limb trauma rats, and further augmented the release of those cytokines and exacerbated lung injury following blast limb trauma. It was reported that NaHS treatment reduced pro-inflammatory cytokines production through inhibiting the activation of NF-kB and P38 MAPK dependent signalling [8], [14].

**Figure 10:**
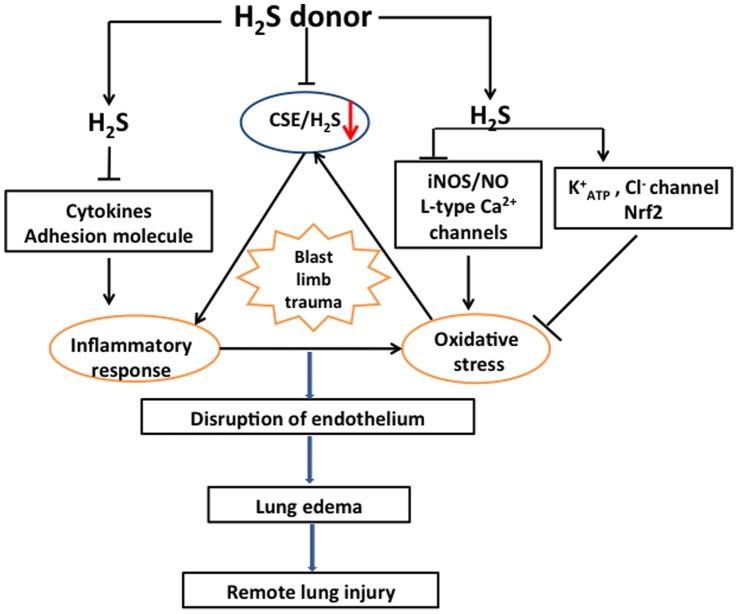
Attenuation of lung injury following blast limb trauma by NaHS treatment. Blast limb trauma induces systemic inflammatory response, oxidative stress and suppression of CSE/H_2_S pathway. All those induce to the disruption of endothelium and the increase of permeability of gas blood barrier, and ultimately lead to lung oedema formation and remote lung injury following blast limb trauma. Exogenous NaHS treatment attenuates lung injury following blast limb trauma via preventing the decrease of plasma H_2_S, suppressing the production of cytokines and adhesion molecular, such as TNF-α, IL-6, IL-10, ICAM1, CXCL-2 and CD11b [31] and ameliorating oxidative stress though Nrf2, K^+^_ATP_ and Cl^−^ channel and inhibition of iNOS/NO pathway and L-type Ca2+ channels [44], [45], [46].

MPO is a heme-containing enzyme within the azurophil granules of neutrophils and the surrogate marker of neutrophils sequestration was used as a quantitative measure of its activity [21]. Infiltration and accumulation of neutrophils promotes the increase of permeability of blood gas barrier, which forms with pulmonary micro-vessel endothelial cells and epithelial cells, and oedema formation [22], [23]. In the present study, NaHS treatment significantly decreased the activation and infiltration of neutrophils which is demonstrated by decrease of MPO activity in both plasma and lungs, and is consistent with the results of histological changes and lung water content after administration of NaHS or PAG. Chemo-attractants and adhesion molecules expressed in endothelial cells play important roles in the interaction of endothelial cell and neutrophil [24], [25]. This interaction ultimately leads to neutrophil infiltration into the lungs and promotes the permeability of the blood gas barrier and subsequently results in lung oedema formation [26]. The interaction of endothelial cell including PMVEC and neutrophil is very complicated whilst the firm neutrophil - PMVEC adhesion is the key step of progression of acute lung injury and mainly mediated by ICAM-1 [27], [28]. Expression of ICAM-1 on endothelial cell is augmented by other cytokines [29], [30]. This is also supported in this study showing that TNF-α induced an increase of ICAM1 expression and IL-6 production in PMVECs which was reduced by NaHS treatment. All those may indicate that NaHS inhibits the interaction of PMVECs and neutrophils at least partially through directly suppression of ICAM-1 expression (Fig. 10). In line with our finding, NaHS or sodium sulphide (Na_2_S), another H_2_S donor, were reported to attenuate leukocyte adherence to endothelium through reduction of expression of the neutrophil chemoattractant cytokine CXCL2, the leukocyte adhesion molecule CD11b and vascular cell adhesion molecule 1 and E, P-selectins [8], [31] and NaHS treatment was shown to directly inhibit the monocyte adhesion to the activated endothelial cells treated with TNF-α *in vitro* [8].

It is well-known that oxidative stress is a fundamental component of pathogenesis of acute lung injury [27], [32], [33]. MDA is the final product of peroxidation. Both SOD and GSH are important protective antioxidant against oxidants and electrophilic compounds, which are shown to be critical to the lungs’ antioxidant defences, particularly in protecting epithelium and endothelium from oxidant injury and inflammation. Blast limb trauma results in a remarkable oxidative stress which is demonstrated by increase of MDA and deplete of SOD and GSH in both plasma and the lungs. Oxidative stress increases the permeability of the blood gas barrier and results in the lung oedema formation [32]. NaHS treatment reported here is shown to alleviate oxidative stress, which is partially contributed to the protection of NaHS against lung injury induced by blast limb trauma. On the other hand, blocking H_2_S production with PAG further exacerbated oxidative stress and lung injury, which is indeed the case shown in our study. The main causes of oxidative stress are likely to be due to the blood loss resulted by limb trauma, free radicals release from active neutrophils and in conjunction with haemoglobin release from erythrocytes [34].

SOD and GSH were reported to be regulated by Nrf2 [35]. Nrf2 is essential in cytoprotection via induction of antioxidant and detoxifying enzymes and proteins via its binding to the cis-acting antioxidant response element (ARE). Nrf2 was shown to protect against lung injury via suppression of oxidative stress and inflammatory reaction [35], [36], [37]. Nrf2 knockdown by siRNA exacerbated the PMVECs injury induced by TNF-α, and this manipulation partially counteracted the protective effect of NaHS shown in this study, which suggests that Nrf2 plays, at least in part, a role in NaHS afforded protection against lung injury. This conclusion can be also supported by up-regulation of Nrf2-mediated downstream effectors such as GSH and heme oxygenase 1 after H_2_S donor treatment reported here and by other’s studies as well [8], [31].

In the present study, NO production induced by TNF-α is shown to be decreased by NaHS treatment due to decrease of iNOS activity in PMVECs. Overproduction of NO leads to oxidative stress though nitrogen species (ROS and RNS) formation, thus promotes the progression of acute lung injury [20], [38]. Mice lacking the iNOS gene showed a mild degree of acute lung injury induced by endotoxin [39], [40]. All those suggest that suppression iNOS/NO pathway may be responsible for the protection of NaHS against lung injury induced by blast limb trauma (Fig. 10). Consistent with our results, the work by Kubo et al also demonstrated that NaHS treatment decreased NO formation via suppressing eNOS and reducing L-arginine transport [41], [42]. In contrast, it was reported that NaHS precondition increased the production of NO or increase of NOS activity in the condition of myocardial ischemia-reperfusion [43]. The causes of this discrepancy are unknown but the different experimental models being used can be one of reasons but the mechanisms of NOS/NO regulated by NaHS under different conditions are needed to further clarify in the future. The suppression of L-type Ca^2+^ channels to maintain Ca^2+^ homeostasis, activating K^+^_ATP_ and CL^−^ channels were reported to be also involved in the protective effects of NaHS (Fig. 10) [44], [45], [46].

NaHS treatment at least partially reversed the decrease of arterial blood pressure induced by blast trauma, which is similar to the results showing that NaHS treatment improves the hemodynamics in rats suffered from hemorrhagic shock, and this effect is considered to be associated with NaHS protection against oxidative stress and inflammatory response [47]. Ultimately, blood perfusion to the vital organs was likely improved and this hemodynamic improvement afforded by NaHS may indirectly contribute to negate lung injury following blast limb trauma. NaHS treatment had no significant effects on the body temperature which is in line with the study reported previously [11].

### Conclusions

We demonstrated that NaHS, an exogenous H_2_S donor, conferred protection against lung injury following blast limb trauma. The mechanisms are likely associated with the suppression of inflammatory response and oxidative stress and Nrf2 cellular signal enhancement.

## Materials and Methods

### In vitro Experiment Protocol

Primary PMVECs were cultured as described previously [5]. Briefly, newborn SD rats were decapitated under 2% isoflurane anaesthesia and their lungs were isolated and then cut into 1×1×1 mm size pieces which were cultured in the plate pre-coated culture with 1% gelatin in a humidified 5% CO_2_ incubator with RPMI-1640 supplemented with 20% fetal bovine serum, 100 U/ml penicillin and streptomycin, 90 u/ml heparin. After 60 hours culture, the tissue were discarded and the reminders of PMVECs were cultured for up to 7–10 days. When reached 60–70% confluence, cells were treated with TNF-α (20 ng/ml or 5 ng/ml) (Millipore, Billerca, MA, USA) alone or superposed with 100 µM or 300 µM NaHS (Sigma-Aldrich, St. Louis, USA) which is equal to the physiological range 18 or 55 µM of H_2_S in body fluid [48]. Cell viability was determined with 3-(4, 5-Dimethyl-2-thiazolyl)-2, 5- diphenyl-2H-tetrazolium bromide (MTT) assay and cell injury was measured with lactic dehydrogenase (LDH) assay in the culture media at 4 h after treatment [13]. iNOS in cell lysates and NO concentration in culture media were determined by the assay kits (Jiancheng biotechnology company, Nanjing, China) followed with the manufacturer’s protocol. ICAM1 and IL 6 in cell lysates were determined with enzyme-linked immunosorbent assay kits (R&D system, Minneapolis, USA) according to the manufacturer’s instructions.

In order to explore the role of Nrf2 on the protection of NaHS against TNF-α induced cell injury, Nrf2 protein expression was knocked-down with Nrf2 siRNA (Santa cruz biotechnology, CA, USA). Briefly, PMVECs were cultured in 6-well plates at a density of 4–5×10^5^ cells per well in RPMI 1640 with 20% FBS without antibiotics. Cells were grown to 50–70% confluence and then transfected with the Nrf2 siRNA together with transfection reagent (Santa cruz biotechnology, CA, USA) (siRNA/Transfection Reagent = 1/1). The viability of transfected PMVECs was determined with MTT assay at 4 h after TNF-α, NaHS, or TNF-α plus NaHS treatment. The total protein was harvested at 48 h post-transfection to determine the Nrf2 protein expression with western blotting. Briefly, the total protein was subjected to SDS-PAGE (4–10% Tris-glycine sodium dodecyl sulfate polyacrylamide SDS–PAGEgels, Invitrogen, Carlsbad, CA) after gel electrophoresis and then proteins were transferred to a polyvinylidene difluoride membranes (Bio-Rad, Hercules, CA) followed by probed with the rabbit anti Nrf2 (1∶1000, Santa Cruz biotechnology, CA, USA) and mouse anti β actin (Abcam, Cambridge, MA).

### Animal Model of Blast Limb Trauma and Treatments

Adult male Sprague-Dawley (SD) rats, weighing 250±25 g (supplied by Flied Surgery Institute, Third Military Medical University, Chongqing, China), were housed in 12 h light-dark conditions with free access to water and standard laboratory chow. Animal procedures were performed strictly in accordance with laboratory animals use guidelines which was approved by the Ethics Committee of Third Military Medical University, Chongqing, China.

All SD rats were anesthetized with intra-peritoneal (I.P.) pentobarbital injection (50 mg/Kg of body weight). The anaesthetic dose was chosen from our pilot studies in which there was no cardiorespiratory suppression while the depth of anaesthesia was sufficient. Local anesthetic infiltration was performed before femoral artery cannulation. Blast limb trauma was induced with chartaceous electricity detonators as described previously [5]. The animals undergoing blast limb trauma were randomly divided into three groups: control: animals were administrated with vehicle (saline) immediately after blast (n = 8); NaHS treatment: animals were further divided into three sub-groups and administrated (i.p.) with three doses of NaHS solution (10 mg/ml in saline): 18****µmol/Kg, 90 µmol/Kg and 180 µmol/Kg immediately after blast, respectively (n = 8 per sub-group); D-L-propargylglycine (PAG) treatment: animals were administrated (i.p.) with 50 mg/kg PAG (Sigma-Aldrich, St. Louis, USA) (40 mg/ml in saline) immediately after blast (n = 8). All animals received the same volume (1 ml) of saline. Another 8 rats undergoing the same procedure but without blast and any treatments served as the Sham control. Arterial blood pressure and rectal temperature were continuously monitored throughout the experiments with PowerLab (AD Instruments, Colorado Springs, CO, USA). Anaesthesia was maintained by I.P. injection of pentobarbital during the experimental period when necessary. At the end of each experiment which lasted for 6 hrs, arterial blood samples were collected for blood gas analysis and harvesting plasma before animal was sacrificed with overdose pentobarbital I.P. injection. Blood plasma was stored at −80°C for subsequent analysis. The right upper and lower lobe of the lung was harvested for histopathological analysis and for measurement of lung water content, respectively. The left lung was harvested and kept in the liquid nitrogen for further use. The plasma H_2_S level, MPO activity, MDA and SOD activity were determined as reported previously [5].

### Histopathological Examination

The lung tissue was fixed in 10% formalin for 24 hrs followed by dehydration and paraffin-wax embedding and then sectioned into 5 µm-thick slices, and stained with hematoxylin and eosin. The microphotography of the lung sections was taken with a light microscope (Olympas, Tokyo, Japan). The severity of acute lung injury (ALI) was scored in a blinded manner as described previously according to categorical degree scoring injury from 0 (minimal or no damage) to 4 (severe damage) of alveolar congestion, hemorrhage, cell infiltration into the airspace or vessel wall, and thickness of the alveolar wall. The mean score of five random areas/section/animal was used for data analysis.

### Lung Water Content

The weight of right lower lobe of the lung was measured after drying with paper tissues and repeated after 72 hrs in a drying oven at 60°C. The lung water content was calculated with the formula as [(Wet lung weight-dry lung weight)/wet lung weight] ×100%.

### Cytokines Concentration in Plasma and Lung Tissue

Tumour necrosis factor α (TNF-α) and interleukin (IL)-6 concentrations in plasma and homogenized lung tissue were analyzed with the enzyme-linked immunosorbent assay kits (R&D system, Minneapolis, USA) [5].

### Glutathione (GSH) Measurement

Lung tissue was homogenized in PBS buffer, and homogenate was centrifuged at 8000 g for 10 minutes. The supernatant or plasma was mixed with reaction buffer at room temperature for 45 minutes. The final product was read at 412 nm and GSH was calculated according to the manufacture’s protocol (Jiancheng biotechnology company, Nanjing, China).

### Statistical Analysis

Data are expressed as mean ± SEM, unless indicated otherwise. Histological injury scoring data was analysed with analysis of variance (ANOVA) followed by Kruskal-Wallis nonparametric test for comparison and then presented as box-and-whisker plot. The rest data were analysed with ANOVA followed by Newman-Keuls comparison. For two group comparisons, the unpaired Student *t* test was used (GraphPad Software, San Diego, CA). A p value <0.05 was considered to be a statistical significance.

## References

[1] Ascherio A, Biellik R, Epstein A, Snetro G, Gloyd S, et al (1995) Deaths and injuries caused by land mines in Mozambique. Lancet 346: 721–724. 7658871 10.1016/s0140-6736(95)91501-x

[2] Korver AJ (1996) Injuries of the lower limbs caused by antipersonnel mines: the experience of the International Committee of the Red Cross. Injury 27: 477–479. 8977832 10.1016/0020-1383(96)00066-6

[3] Owens JG (2010) Physical therapy of the patient with foot and ankle injuries sustained in combat. Foot Ankle Clin 15: 175–186. 20189123 10.1016/j.fcl.2009.10.005

[4] Champion HR, Holcomb JB, Young LA (2009) Injuries from explosions: physics, biophysics, pathology, and required research focus. J Trauma 66: 1468–1477; discussion 1477. 10.1097/TA.0b013e3181a27e7f 19430256

[5] Ning JL, Mo LW, Lu KZ, Lai XN, Wang ZG, et al (2012) Lung injury following lower extre,ity blast trauma in rats. J Trauma 73: 1537–1544. 10.1097/TA.0b013e318266013a 23064609

[6] Qu K, Lee SW, Bian JS, Low CM, Wong PT (2008) Hydrogen sulfide: neurochemistry and neurobiology. Neurochem Int 52: 155–165. 17629356 10.1016/j.neuint.2007.05.016

[7] Wagner CA (2009) Hydrogen sulfide: a new gaseous signal molecule and blood pressure regulator. J Nephrol 22: 173–176. 19384833

[8] Pan LL, Liu XH, Gong QH, Wu D, Zhu YZ (2011) Hydrogen sulfide attenuated tumor necrosis factor-alpha-induced inflammatory signaling and dysfunction in vascular endothelial cells. PLoS One 6: e19766. 21572963 10.1371/journal.pone.0019766 PMC3091882

[9] Kimura H, Shibuya N, Kimura Y (2012) Hydrogen sulfide is a signaling molecule and a cytoprotectant. Antioxid Redox Signal. 10.1089/ars.2011.4345 PMC3342561 22229673

[10] Aslami H, Heinen A, Roelofs JJ, Zuurbier CJ, Schultz MJ, et al (2010) Suspended animation inducer hydrogen sulfide is protective in an in vivo model of ventilator-induced lung injury. Intensive Care Med 36: 1946–1952. 20721529 10.1007/s00134-010-2022-2 PMC2952106

[11] Faller S, Ryter SW, Choi AM, Loop T, Schmidt R, et al (2010) Inhaled hydrogen sulfide protects against ventilator-induced lung injury. Anesthesiology 113: 104–115. 20574227 10.1097/ALN.0b013e3181de7107

[12] Wang C, Wang HY, Liu ZW, Fu Y, Zhao B (2011) Effect of endogenous hydrogen sulfide on oxidative stress in oleic acid-induced acute lung injury in rats. Chin Med J (Engl) 124: 3476–3480. 22340161

[13] Hinder F, Stubbe HD, Van Aken H, Waurick R, Booke M, et al (1999) Role of nitric oxide in sepsis-associated pulmonary edema. Am J Respir Crit Care Med 159: 252–257. 9872846 10.1164/ajrccm.159.1.9806024

[14] Tokuda K, Kida K, Marutani E, Crimi E, Bougaki M, et al (2012) Inhaled hydrogen sulfide prevents endotoxin-induced systemic inflammation and improves survival by altering sulfide metabolism in mice. Antioxid Redox Signal 17: 11–21. 22221071 10.1089/ars.2011.4363 PMC3342565

[15] Khan AA, Schuler MM, Prior MG, Yong S, Coppock RW, et al (1990) Effects of hydrogen sulfide exposure on lung mitochondrial respiratory chain enzymes in rats. Toxicol Appl Pharmacol 103: 482–490. 2160136 10.1016/0041-008x(90)90321-k

[16] Bhatia M, Wong FL, Fu D, Lau HY, Moochhala SM, et al (2005) Role of hydrogen sulfide in acute pancreatitis and associated lung injury. FASEB J 19: 623–625. 15671155 10.1096/fj.04-3023fje

[17] Tamizhselvi R, Moore PK, Bhatia M (2008) Inhibition of hydrogen sulfide synthesis attenuates chemokine production and protects mice against acute pancreatitis and associated lung injury. Pancreas 36: e24–31. 18437075 10.1097/MPA.0b013e31816857bb

[18] Wintner EA, Deckwerth TL, Langston W, Bengtsson A, Leviten D, et al (2010) A monobromobimane-based assay to measure the pharmacokinetic profile of reactive sulphide species in blood. Br J Pharmacol 160: 941–957. 20590590 10.1111/j.1476-5381.2010.00704.x PMC2936000

[19] Stephens KE, Ishizaka A, Larrick JW, Raffin TA (1988) Tumor necrosis factor causes increased pulmonary permeability and edema. Comparison to septic acute lung injury. Am Rev Respir Dis 137: 1364–1370. 3059859 10.1164/ajrccm/137.6.1364

[20] Yang YL, Huang KL, Liou HL, Chen HI (2008) The involvement of nitric oxide, nitric oxide synthase, neutrophil elastase, myeloperoxidase and proinflammatory cytokines in the acute lung injury caused by phorbol myristate acetate. J Biomed Sci 15: 499–507. 18283562 10.1007/s11373-008-9238-y

[21] Knight KR, Zhang B, Morrison WA, Stewart AG (1997) Ischaemia-reperfusion injury in mouse skeletal muscle is reduced by N omega-nitro-L-arginine methyl ester and dexamethasone. Eur J Pharmacol 332: 273–278. 9300260 10.1016/s0014-2999(97)01101-1

[22] Abraham E (2003) Neutrophils and acute lung injury. Crit Care Med 31: S195–199. 12682440 10.1097/01.CCM.0000057843.47705.E8

[23] Chignard M, Balloy V (2000) Neutrophil recruitment and increased permeability during acute lung injury induced by lipopolysaccharide. Am J Physiol Lung Cell Mol Physiol 279: L1083–1090. 11076798 10.1152/ajplung.2000.279.6.L1083

[24] Bhatia M, Brady M, Zagorski J, Christmas SE, Campbell F, et al (2000) Treatment with neutralising antibody against cytokine induced neutrophil chemoattractant (CINC) protects rats against acute pancreatitis associated lung injury. Gut 47: 838–844. 11076884 10.1136/gut.47.6.838 PMC1728153

[25] Donnelly SC, Haslett C, Dransfield I, Robertson CE, Carter DC, et al (1994) Role of selectins in development of adult respiratory distress syndrome. Lancet 344: 215–219. 7518025 10.1016/s0140-6736(94)92995-5

[26] Eiermann GJ, Dickey BF, Thrall RS (1983) Polymorphonuclear leukocyte participation in acute oleic-acid-induced lung injury. Am Rev Respir Dis 128: 845–850. 6638672 10.1164/arrd.1983.128.5.845

[27] Folch E, Salas A, Panes J, Gelpi E, Rosello-Catafau J, et al. (1999) Role of P-selectin and ICAM-1 in pancreatitis-induced lung inflammation in rats: significance of oxidative stress. Ann Surg 230: 792–798; discussion 798–799. 10.1097/00000658-199912000-00008 PMC1420943 10615934

[28] Lee WL, Downey GP (2001) Neutrophil activation and acute lung injury. Curr Opin Crit Care 7: 1–7. 11373504 10.1097/00075198-200102000-00001

[29] Nathens AB, Bitar R, Watson RW, Issekutz TB, Marshall JC, et al (1998) Thiol-mediated regulation of ICAM-1 expression in endotoxin-induced acute lung injury. J Immunol 160: 2959–2966. 9510200

[30] van Buul JD, van Rijssel J, van Alphen FP, Hoogenboezem M, Tol S, et al (2010) Inside-out regulation of ICAM-1 dynamics in TNF-alpha-activated endothelium. PLoS One 5: e11336. 20596527 10.1371/journal.pone.0011336 PMC2893160

[31] Francis RC, Vaporidi K, Bloch KD, Ichinose F, Zapol WM Protective and Detrimental Effects of Sodium Sulfide and Hydrogen Sulfide in Murine Ventilator-induced Lung Injury. Anesthesiology 115: 1012–1021. 10.1097/ALN.0b013e31823306cf PMC3752661 21912243

[32] Ward PA (2010) Oxidative stress: acute and progressive lung injury. Ann N Y Acad Sci 1203: 53–59. 20716283 10.1111/j.1749-6632.2010.05552.x

[33] Lange M, Szabo C, Traber DL, Horvath E, Hamahata A, et al (2012) Time profile of oxidative stress and neutrophil activation in ovine acute lung injury and sepsis. Shock 37: 468–472. 22266977 10.1097/SHK.0b013e31824b1793 PMC4646062

[34] Vega VL, Maldonado M, Mardones L, Schulz B, Manriquez V, et al (1999) Role of Kupffer cells and PMN leukocytes in hepatic and systemic oxidative stress in rats subjected to tourniquet shock. Shock 11: 403–410. 10454829

[35] Cho HY, Kleeberger SR (2010) Nrf2 protects against airway disorders. Toxicol Appl Pharmacol 244: 43–56. 19646463 10.1016/j.taap.2009.07.024

[36] Chan K, Kan YW (1999) Nrf2 is essential for protection against acute pulmonary injury in mice. Proc Natl Acad Sci U S A 96: 12731–12736. 10535991 10.1073/pnas.96.22.12731 PMC23072

[37] Papaiahgari S, Yerrapureddy A, Reddy SR, Reddy NM, Dodd OJ, et al (2007) Genetic and pharmacologic evidence links oxidative stress to ventilator-induced lung injury in mice. Am J Respir Crit Care Med 176: 1222–1235. 17901416 10.1164/rccm.200701-060OC PMC2176106

[38] Su CF, Yang FL, Chen HI (2007) Inhibition of inducible nitric oxide synthase attenuates acute endotoxin-induced lung injury in rats. Clin Exp Pharmacol Physiol 34: 339–346. 17324147 10.1111/j.1440-1681.2007.04553.x

[39] Hesse AK, Dorger M, Kupatt C, Krombach F (2004) Proinflammatory role of inducible nitric oxide synthase in acute hyperoxic lung injury. Respir Res 5: 11. 15377396 10.1186/1465-9921-5-11 PMC520822

[40] Peng X, Abdulnour RE, Sammani S, Ma SF, Han EJ, et al (2005) Inducible nitric oxide synthase contributes to ventilator-induced lung injury. Am J Respir Crit Care Med 172: 470–479. 15937288 10.1164/rccm.200411-1547OC PMC2718528

[41] Kubo S, Doe I, Kurokawa Y, Nishikawa H, Kawabata A (2007) Direct inhibition of endothelial nitric oxide synthase by hydrogen sulfide: contribution to dual modulation of vascular tension. Toxicology 232: 138–146. 17276573 10.1016/j.tox.2006.12.023

[42] Kubo S, Kurokawa Y, Doe I, Masuko T, Sekiguchi F, et al (2007) Hydrogen sulfide inhibits activity of three isoforms of recombinant nitric oxide synthase. Toxicology 241: 92–97. 17888559 10.1016/j.tox.2007.08.087

[43] Yong QC, Lee SW, Foo CS, Neo KL, Chen X, et al (2008) Endogenous hydrogen sulphide mediates the cardioprotection induced by ischemic postconditioning. Am J Physiol Heart Circ Physiol 295: H1330–H1340. 18660450 10.1152/ajpheart.00244.2008

[44] Mikami Y, Shibuya N, Kimura Y, Nagahara N, Yamada M, et al (2011) Hydrogen sulfide protects the retina from light-induced degeneration by the modulation of Ca2+ influx. J Biol Chem 286: 39379–39386. 21937432 10.1074/jbc.M111.298208 PMC3234762

[45] Kimura Y, Dargusch R, Schubert D, Kimura H (2006) Hydrogen sulfide protects HT22 neuronal cells from oxidative stress. Antioxid Redox Signal 8: 661–670. 16677109 10.1089/ars.2006.8.661

[46] Kimura Y, Goto Y, Kimura H (2010) Hydrogen sulfide increases glutathione production and suppresses oxidative stress in mitochondria. Antioxid Redox Signal 12: 1–13. 19852698 10.1089/ars.2008.2282

[47] Ganster F, Burban M, de la Bourdonnaye M, Fizanne L, Douay O, et al (2010) Effects of hydrogen sulfide on hemodynamics, inflammatory response and oxidative stress during resuscitated hemorrhagic shock in rats. Critical Care 14: R165. 20836847 10.1186/cc9257 PMC3219260

[48] Dombkowski RA, Russell MJ, Olson KR (2004) Hydrogen sulfide as an endogenous regulator of vascular smooth muscle tone in trout. Am J Physiol Regul Integr Comp Physiol 286: R678–685. 15003943 10.1152/ajpregu.00419.2003

